# Risk Assessment of Dynamic Diffusion of Urban Non-Point Source Pollution Under Extreme Rainfall

**DOI:** 10.3390/toxics13050385

**Published:** 2025-05-09

**Authors:** Ting Wen, Chuanxun Li, Jiawen Liu, Peng Wang

**Affiliations:** Faculty of Civil Engineering and Mechanics, Jiangsu University, Zhenjiang 212013, China; upesawt@stmail.ujs.edu.cn (T.W.); lichuanxun@yeah.net (C.L.); upesaljw@stmail.ujs.edu.cn (J.L.)

**Keywords:** cellular automata, export coefficient model, extreme rainfall, land use, non-point source of pollution, risk assessment

## Abstract

With the acceleration of urbanization, the diffusion mechanism of urban non-point source (NPS) pollution caused by extreme rainfall is not clear, which leads to high cost and difficulty in water environment treatment. In view of the shortcomings of dynamic diffusion simulations of mesoscale pollution, this paper proposes a simulation framework based on cellular automata, GIS geographic technology, and a two-dimensional shallow water model. Taking the 500 m × 500 m grid as the unit, we explore the migration laws of nitrogen and phosphorus pollutants and the response relationship between pollutant diffusion and land use under extreme rainfall scenarios. The results show that (i) the pollution risk increases significantly with diffusion, with the maximum pollution load in high-risk areas increasing by 181%, and the diffusion rate is positively correlated with the rate of change in rainfall intensity; (ii) forest land has the highest grid pollution load loss rate, whereas the water grid has the highest accumulation rate; (iii) this method can accurately identify the hot spots of pollution diffusion, providing a basis for the precise control of high-risk areas. This study can support the targeted governance of pollution sources and land planning optimization in urban storm and flood management, and help reduce environmental health risks in extreme climates.

## 1. Introduction

The surface runoff formed during heavy rains brings many pollutants into water bodies in a short period of time, causing serious non-point source (NPS) pollution and adversely affecting the quality of the urban water environment [1,2]. As urbanization accelerates, the natural permeable ground in urban regions continues to decrease, the impermeable underlying surface continues to increase, and the process of surface runoff creation speeds up [3,4], further exacerbating the risk of NPS pollution. Furthermore, NPS pollution has also been significantly impacted by changes in land use, particularly the expansion of downtown areas and the elimination of natural spaces [5,6,7]. As a result, NPS pollution caused by rainfall is now the primary source of the decline in urban water quality [8]. The resulting pollution risks have a negative impact on the urban natural environment and human well-being [9]. Due to the complex characteristics of NPS pollutants, numerous influencing factors (such as land use, rainfall intensity, topography, etc.), and diverse types of urban surface coverage, the distribution of pollution is difficult to clarify, making it difficult and costly to control NPS pollution incidents [10,11]. Consequently, investigating the urban NPS pollution risk aggravating process after heavy rainfall is essential to develop a theoretical framework for urban water pollution mitigation [12,13].

Previous studies on NPS pollution in cities under extreme rainfall have been conducted from multiple perspectives. Regarding simulation methods, previous studies mainly used two categories: lumped models (mainly empirical models) and distributed models (physical models and GIS-based hydrological models). Lumped models like the Long-Term Hydrologic Impact Assessment (L-THIA) reduce internal spatial heterogeneity, address the research region as a whole, and improve computational efficiency by simplifying the model structure [14,15]. Distributed models such as the Soil and Water Assessment Tool (SWAT), Water Erosion Prediction Project (WEPP) model, Storm Water Management Model (SWMM), etc., can map the spatiotemporal distribution characteristics of NPS pollution loads and more clearly describe the spatial heterogeneity of pollution distribution [16,17]. They divide the watershed into multiple sub-units (such as sub-watersheds, grids, etc.) for separate simulations and then summarize them to obtain the overall results [18,19], so that the simulated pollutant migration and diffusion process is more accurate. From a research scale perspective, there are both macro-scale studies and micro-scale studies. Macro-scale studies mainly focus on the pollution load and pollutant migration and transformation processes in watersheds or urban areas [11,20], while micro-scale studies are primarily concerned with the identification of pollution sources, pollutant production, and emission mechanisms within a single plot or block [21,22,23].

However, there are still some limitations of previous research. With reference to the simulation methodology, lumped models can obtain an overall quantitative relationship but it is difficult to reflect the spatial distribution characteristics of pollution [24,25]. The implementation of a distributed model requires a large amount of data, and many data are difficult to obtain, which limits its application scope [26,27]. In this issue, cellular automata (CA) models have been proven to streamline the required parameter data while accurately simulating contaminant diffusion [28,29]. The CA model can realize complex spatiotemporal dynamic simulation of pollutant migration and diffusion [30]. The model is founded upon cellular bases, each of which represents a specific area. Depending on the research area, the size of the basic cell varies. The size-related constraints of most existing studies can be successfully addressed by adjusting the cell size in CA. In addition, through flexible modification of the CA model generation program to consider the influence of rainfall intensity, terrain, land use type, and other parameters, the simulation results can be brought closer to reality. At the same time, the CA model can be integrated well with GIS and other distributed hydrological models, thereby enabling multi-dimensional analysis to make spatial analysis more powerful and intuitive and clearly display the time and geographical dispersion results of NPS pollution [31,32,33].

In terms of the research scale, macro-scale research cannot accurately describe the detailed characteristics of the pollution process [34], while micro-scale research is difficult to analyze the overall environmental change trend. In addition, due to limitations in research scale, when studying the impact of urban land use changes on pollutant diffusion, more research focuses on the correlation between the overall land use amount and pollutant diffusion, while there is less research on the impact of various land types on pollutant diffusion [35,36,37]. In solving the problem of research scale limitations, a grid scale of 500 m × 500 m is considered to be appropriate when using cellular automata to simulate pollutant diffusion. It is neither too microscopic to analyze the overall environmental change trend nor too macroscopic to ignore local pollution details. Compared with the micro scale, this scale makes it easier to obtain geographical data such as terrain and land use, does not require too much detailed data, and can reduce a certain number of samples to reduce the model running time and running costs [38]. Compared with macro scales such as urban or watershed scales, this scale can more accurately reflect the spatial differences in each research plot, thereby more accurately representing the process of migration and diffusion of NPS pollution. Regarding the management and prevention in urban areas, this scale is more conducive to risk warning and regulation for high-risk areas and potential risk areas [32,39,40].

In order to make up for the limitations of previous research, this study uses the cellular automata (CA) model combined with GIS technology to simulate NPS pollution in urban areas under rainfall scenarios. The cell size used in this investigation was 500 m by 500 m, which is a medium scale. In addition, a two-dimensional shallow water model accelerated by a general-purpose graphics processing unit (SW2D-GPU) model was also coupled with the CA model to simulate extreme rainfall scenarios as a prerequisite rainfall intensity condition for urban NPS pollution simulations [41,42]. The primary goal of this research is to propose a risk analysis method for urban NPS pollution transport and diffusion under a medium-scale extreme rainfall, and to investigate the effects of urban land utilization upon pollutant diffusion. Regarding the current situation of increasing urban NPS pollution caused by extreme rainfall, the findings can furnish technical assistance in pinpointing pivotal pollution hotspots and provide a theoretical basis for rationally regulating land use attributes to mitigate the impact of NPS pollution.

## 2. Materials and Methods

**Figure 1:**
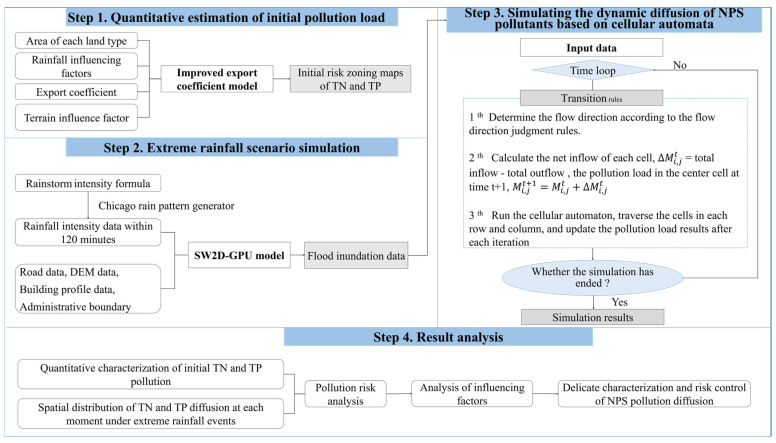
Methodology framework.

The research subjects were nitrogen and phosphorus nutrients, which are major pollutants in NPS pollution. To replicate the larger-scale dynamic diffusion of total nitrogen (TN) and total phosphorus (TP), the study area was subdivided into 676 grids, each measuring 500 m by 500 m. This study first uses the export coefficient model to quantify the initial pollution load of TN and TP, and implements risk zonation through ArcGIS to obtain the initial pollution risk zoning maps. Next, a pollution dynamic diffusion simulation model was constructed based on cellular automation and the SW2D-GPU model. This study used this model to replicate the continual diffusion of NPS pollution under an extreme rainfall scenario that occurs once every 1000 years, and used the simulation result to analyze the pollution risk changes of TN and TP. Figure 1 is an illustration of the methodology framework.

### 2.1. Quantitative Estimation of Initial Pollution Load

We evaluated the initial pollutant load (L) using the modified Johnes export coefficient model [14,43]. The following is the calculating formula:

(1) $$L=X\cdot Y\sum_{i=1}^{n}E_{i}\left[A_{i}\left(I_{i}\right)\right]+p,$$

The rainfall influence factor is represented by X, and the geographic influence factor by Y. The i-th land use type’s output coefficient is denoted by $E_{i}$ (kg/(ha∙a)), which represents the mass of pollutant output per unit area of land per year. The area of the i-th land use type is denoted by $A_{i}$ (m^2^), the input intensity of the various land use types is indicated by $I_{i}$ (kg/a), and the loads generated by atmospheric deposition in the research region is denoted by *p* (kg/a). Since the input pollutant mass p from precipitation is very small, the influence of this factor was not considered.

Coefficients are used to quantify the contribution of different land use types to non-point source pollution (NPS), and these coefficients reflect the amount of pollutant output per unit area generated by a particular land use type. Combined with the geographical characteristics of the watershed, the pollution export coefficients ($E_{i})$ were obtained by consulting the literature; the export coefficients were determined as shown in Table 1. Based on the geographical characteristics of the watershed, we searched the literature to obtain the pollution export coefficients ($E_{i})$ [44]. The export coefficients are shown in Table 1.

### 2.2. Extreme Rainfall Scenario Simulation with a Recurrence Period of 1000 Years

**Figure 2:**
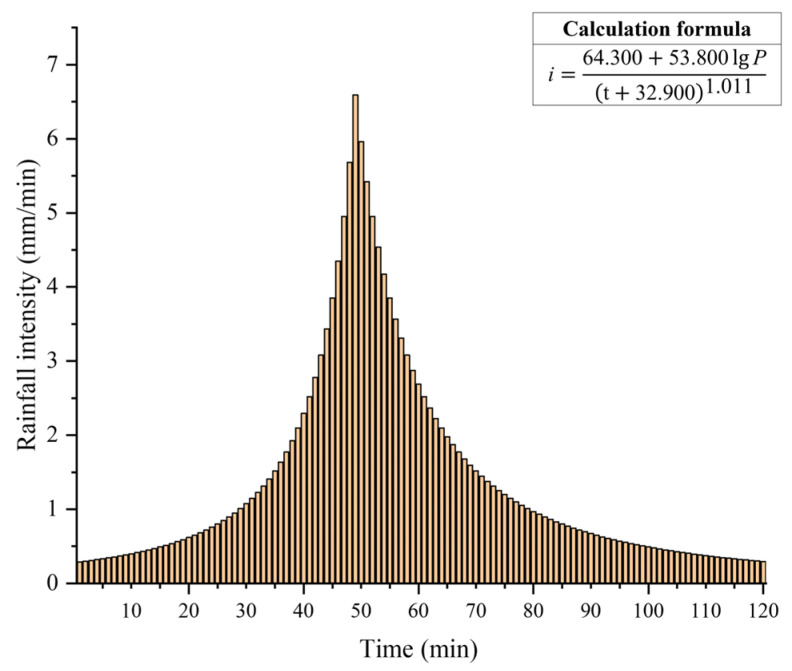
Rainfall intensity under a 1000-year recurrence period.

This study calculates rainfall intensity based on the revised Nanjing rainstorm intensity formula and outdoor drainage design standards [45]. In addition, since this study needs to examine the pollution risk under extreme rainfall scenarios, the recurrence period is set to 1000 years and the simulation duration is 120 min. Figure 2 represents the calculated rainfall intensity data within 120 min. And, then, the Chicago rain pattern generator is used to form the corresponding precipitation distribution. Software such as VISUAL STUDIO and CUDA/C++ are adopted to run the SW2D-GPU model to generate extreme rainfall scenarios [1]. In light of the simulation’s findings, flood depth, flow velocity, flow direction, and other inundation data of the study area at each time within 120 min are obtained, which provides a data basis for subsequent simulation of the dynamic diffusion process of TN and TP.

### 2.3. Simulating the Dynamic Diffusion of NPS Pollution Based on Cellular Automata

**Figure 3:**
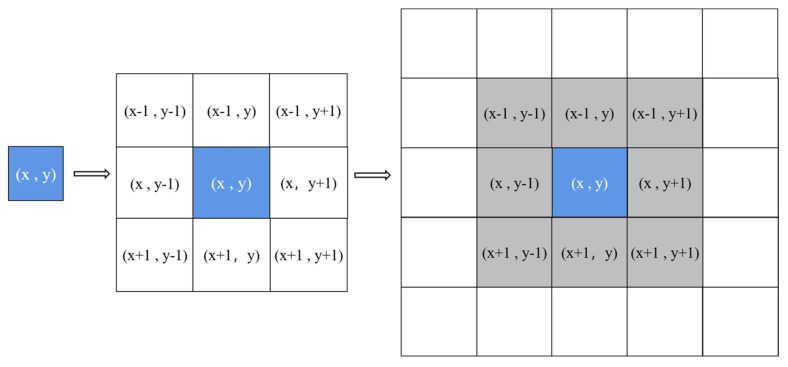
Moore-type neighborhood and cellular space composition.

The cellular automaton model (cellular automata, CA) is a dynamic model that is discrete in time and space. CA mainly includes four parts: cellular space, neighborhood, state, and rules [29]. The cellular rules are the core of CA, which mainly define the rules for the evolution of the cell from the current state to the next moment and determine how to perform local transformations between cells. The cell neighborhood adopts a typical Moore-type neighborhood, as shown in Figure 3.

To simulate pollutant diffusion process, this study split the research region into 500 m × 500 m grid cells and defined contaminant transmission rules between cells and neighboring units or neighborhoods in CA. We assumed that there is no chemical reaction between pollutants and water, and only changes in the mass of pollutants are considered. The diffusion is only affected by factors such as water flow direction, flow velocity, and flow rate, and satisfies the principle of mass conservation.

First, considering the water flow velocity and the grid cell area of 500 × 500 m, when the iteration time step is set to 5 min, the pollutant diffusion conversion between local areas is more complete. After setting the local conversion rules for the output of the central cell to the surrounding cells and the input of the surrounding cells, the parameter data such as water flow and pollutant quality required in the diffusion process are imported. According to the diffusion rules, the amounts of pollutants transferred from the central cell to the surrounding cells are allocated, and, then, the state of TN and TP in the cell is updated at the next moment. After iterating a certain number of times, the final diffusion result is obtained.

Second, the cell conversion rule is to determine flow direction between the core cell and the surrounding eight cells through assessing water level, and then judge flow direction according to the area with a high water level flowing to the area with a low water level.

**Figure 4:**
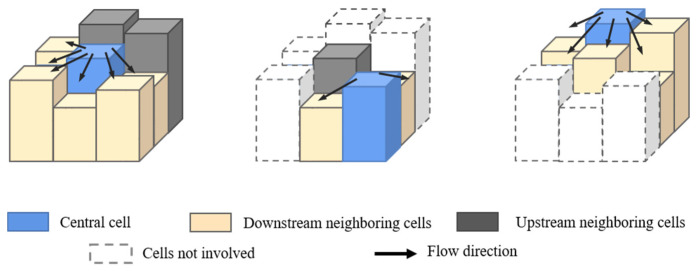
Flow judgment rules.

The flow direction of cells is determined based on the rule that cells with high water levels flow to cells with low water levels [46]. The flow direction judgment is shown in Figure 4.

(2) $$H_{i,j}^{t}=h_{i,j}+d_{i,j}^{t},$$

where $H_{i,j}^{t}$ is the water level height (m) of the cell in the *i*-th row and *j*-th column at time *t*, $h_{i,j}$ is the terrain elevation value of the cell in the *i*-th row and *j*-th column (m), and $d_{i,j}^{t}$ is the water depth (m) in the cell of the *i*-th row and *j*-th column at time *t*.

Finally, to determine the amount of pollutants carried away at each moment, the rainfall intensity already determined is employed. Predicated on the idea that the quantity of water flow and pollution transmission are proportionate [29,47], the existing water flow diffusion data are used as a benchmark to calculate the amount of pollution transfer. The previously simulated rainfall data are obtained for this study, and based on the changing trend of the water flow data at the present time and the water flow data at subsequent times in each central cell, the inflow and outflow of pollutants between the central cell and neighboring cells are calculated.

Within the unit time step, when shifting to the horizontal/vertical direction, the formula for the mass of pollutants flowing from the central cell to neighboring cells $\Delta M_{i,j}^{t}$ is as listed below:

(3) $$\Delta M_{i,j}^{t}=\frac{M_{i,j}^{t}\times {v_{X}}_{i,j}^{t}\left({v_{Y}}_{i,j}^{t}\right)\times t_{s}}{b}$$

When shifting diagonally, the mass of pollutants flowing from the core cell to the surrounding cells $\Delta M_{i,j}^{t}$ is as listed below:

(4) $$\Delta M_{i,j}^{t}=\frac{M_{i,j}^{t}\times \sqrt{({{v_{X}}_{i,j}^{t})}^{2}+({{v_{Y}}_{i,j}^{t})}^{2}}\times t_{s}}{\sqrt{2}b}$$

where $M_{i,j}^{t}$ is the mass of pollutants contained in the core cell at time t (kg), ${v_{X}}_{i,j}^{t}$, ${v_{Y}}_{i,j}^{t}$ are flow velocity (m/s) in the x and y directions, $t_{s}$ is time step (s), and b is side length of the grid unit (m).

### 2.4. Case Description

**Figure 5:**
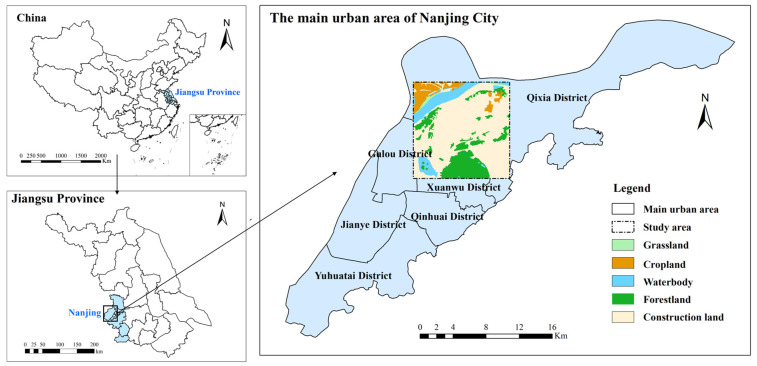
Study area.

The research area is situated within the main urban zone of Nanjing City, Jiangsu Province, China. Since most of the main urban areas are urban construction land and the categories of land usage are relatively single, we selected the area with the largest number of land use types as the research area (Figure 5). The watershed covers an area of 169 square kilometers, covering 5 land use types: cropland, grassland, forest land, construction land, and water bodies.

The fundamental data needed are land cover statistics (30 m × 30 m), DEM data (30 m × 30 m), and 44 years of Nanjing meteorological station rainfall monitoring data. Among them, the 30 m resolution data are resampled and summarized into a 500 m grid through ArcGIS, and spatial heterogeneity is preserved through the average method. In this study, land cover statistics in Nanjing produced based on Landsat remote sensing images were obtained through commercial procurement. The rainfall meteorological data were obtained through commercial procurement, and the data source provider is the National Meteorological Information Center. This data set contains daily rainfall observation records of various meteorological sites in Nanjing from 1979 to 2022. The above data are critical for quantitative estimates of pollutant load. For the rainfall scenario simulation module, more detailed data are needed, including road network data, building profile data and administrative boundaries, etc. These are obtained through BIGEMAP (www.bigemap.com).

### 2.5. Sensitivity Analysis of Extreme Rainfall Simulation Results

Previous studies [48] have validated the SW2D-GPU model through case studies involving urban flood simulations and lake water level simulations, including case validation, numerical stability validation, and parallel performance validation, ensuring the accuracy of the model for urban flood simulations. This prior validation confirms the applicability of the SW2D-GPU model for urban flood modeling. To further investigate the stability and reliability of the model under different conditions, this study assesses the robustness of key parameters involved in the model. The parameters validated include Manning’s roughness coefficient, permeability coefficient, iterative computation time step, and grid refinement level.

By systematically varying the values of each parameter and analyzing their impact on the model outcomes, we aimed to understand the model’s stability in response to parameter fluctuations. The baseline scenario parameters are listed in Table 2. The SW2D-GPU model was executed sequentially with these parameters to obtain model outputs, and the inundation extent and maximum depth of the study area at the 120th min were compared. The results are summarized in Table 2.

Analysis of the simulation results obtained by adjusting each target parameter individually revealed that grid refinement has a more significant impact on the accuracy of the model’s simulation results. To ensure high accuracy in the model’s simulation outcomes, this study considered the appropriate grid refinement level for the study area’s size and selected a 5 m grid data for analysis. The 5 m data were then aggregated into a 500 m grid network using ArcGIS (Version 10.8) to facilitate subsequent data analysis at 500 m grid cell units. Given the robustness of the other three parameters, it can be concluded that the model results in this study are stable and reasonable, providing a reliable data foundation for subsequent analyses.

## 3. Results

### 3.1. Quantitative Characterization of Initial NPS Pollution

**Figure 6:**
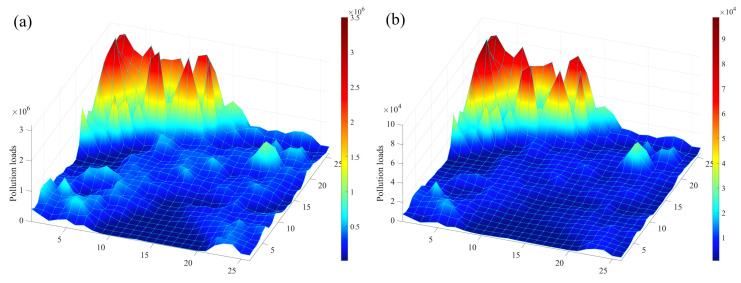
Initial pollution risk zoning of TN (**a**) and TP (**b**).

Since there is currently no unified standard for pollutant risk zoning, the natural breakpoint method in ArcGIS is used for risk zoning, and the risk is divided into five levels, which is extremely low-risk, low risk, medium risk, high risk, and extremely high risk. This method was applied to the pollution load data to generate the initial pollution risk zoning maps. Figure 6 shows the initial pollution risk zoning map, and the pollution load indicates the level of pollution risk. Table 3 and Table 4 show the specific pollution risk zonation. The pollutants have similar characteristics in that the areas with the largest proportion of risks are extremely low-risk areas and low-risk areas, while medium-risk, high-risk, and extremely high-risk areas have a comparatively small percentage of area. Comparing the quantity of pollution load, the pollution load in the extremely high-risk areas is much greater than that in the low-risk areas.

### 3.2. Analysis of Risk Changes in NPS Pollution Under Extreme Rainfall Senarios

**Figure 7:**
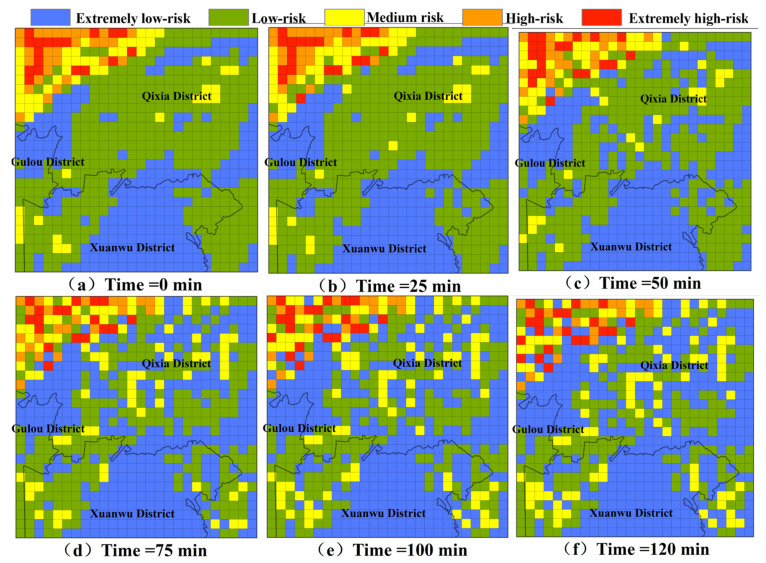
The pollution risk changes in TN under extreme rainfall scenarios.

**Figure 8:**
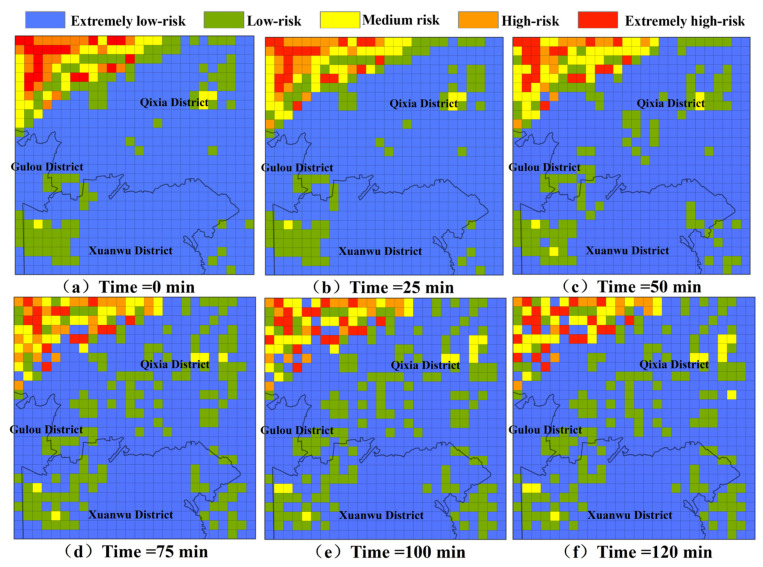
The pollution risk changes in TP under extreme rainfall scenarios.

Figure 7 and Figure 8 present the pollution risk changes in NPS-TN and NPS-TP under extreme rainfall scenarios, which are the results simulated using cellular automata, and the selected moments for analysis are the 0th, 25th, 50th, 75th, 100th, and 120th min, respectively. The pollution risk zoning range in the dynamic diffusion process is set with reference to the risk zoning range in the initial pollution state. It is evident that the risk change trends of TN and TP are generally consistent. The pollution risk zoning maps have changed greatly: more and more medium-risk grids appear in the low-risk area of TN, and more and more low-risk grids appear in the extremely low-risk area of TP. At the same time, high-risk areas are more dispersed and the number of grids increases. From the 0th min to the 120th min, the risk change trend first becomes larger and then becomes smaller. Among them, the pollution risk changes most significantly from the 25th min to the 50th min and from the 50th min to the 75th min.

**Figure 9:**
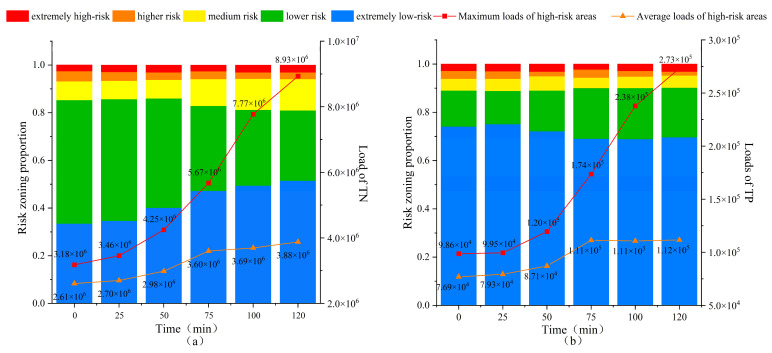
Risk zoning and pollution load changes in TN (**a**) and TP (**b**).

Figure 9 shows the area proportion of each risk area in TN and TP and the pollution load changes in extremely high-risk areas. The areas of extremely low-risk, medium-risk, and extremely high-risk all show an upward trend. Among them, the extremely low-risk area has the highest growth rate (increasing from 33.48% to 51.48%), and the extremely high-risk area shows a weak growth trend in area (increasing from 2.67% to 3.10%). Judging from the changing trend of the maximum pollution load, although the area of the extremely high-risk area has not increased much, its maximum pollution load has increased from 3,177,390.51 to 8,932,640.19 kg. The increased pollution risk is extremely serious, and the growth trend first became larger and then flattened. Judging from the average risk of the extremely high-risk area, it shows a clear upward trend, increasing from 2,605,548.3 to 3,875,402.604 kg. However, compared with the maximum pollution load, the growth trend is relatively gentle.

**Figure 10:**
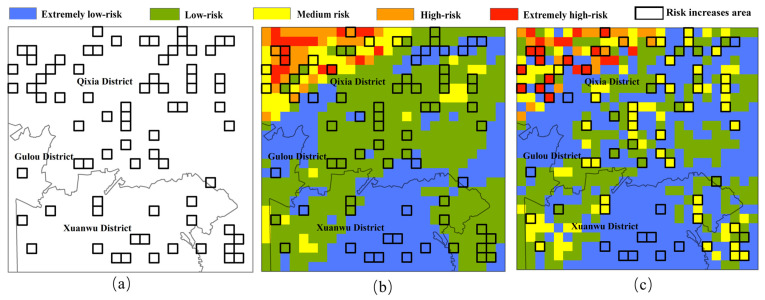
Areas where pollution risks increase too much (**a**); risk distribution before spread (**b**); risk distribution after spread (**c**).

Compared with previous studies, this study combines the cellular automaton model to characterize the local migration and diffusion process of pollutants on the basis of characterizing static pollution risks, and evaluates the dynamic risks of surface pollutants under extreme rainfall conditions. Based on the characterization of dynamic risks, grid areas with excessive load growth during the pollution diffusion process can be screened out from the risk maps of TN and TP, thereby providing technical guidance and governance suggestions for precise control of areas prone to pollution. As shown in Figure 10, in this simulation, TN is used as an example to accurately locate grids with excessive pollution load growth. It can be seen that after the diffusion process of risk partitions in these grid areas, the pollution risk has greatly increased.

### 3.3. Spatial Correlation Characteristics Between NPS Pollutant Diffusion and Land Use Attributes

**Figure 11:**
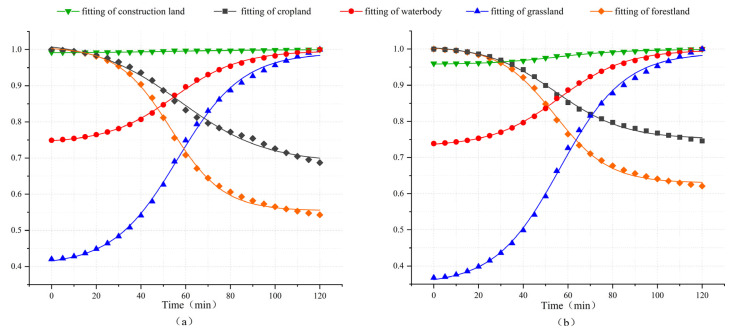
Fitted curves of average load in each land use type of TN (**a**) and TP (**b**).

During the dynamic diffusion process of NPS pollution, since the time scale studied in this study is small and land changes are negligible, the emphasis is on the spatial correlation characteristics between NPS pollutant diffusion and land use attributes. The MAXs method among the dimensionless processing methods is used to process average pollution load data; then, the data are fitted to a curve as shown in Figure 11, and analysis is performed on how different land use types affect the dynamic diffusion of NPS pollutants.

Based on the general pattern, the pollution load in cropland and forest land continues to decrease, the pollution load in water bodies and grassland continues to increase, while the pollution load changes in construction land are not significant. From the perspective of the change rate, the pollution load loss rate in forest land is higher than that in cultivated land, and the pollution load accumulation rate in grassland is higher than that in water bodies. However, analyzing the average pollution load can only provide a general change trend, and further analysis of local changes in different land use categories is required.

## 4. Discussion

In this study, we specifically focus on the diffusion of TN and TP, which are major pollutants in urban NPS pollution. The sources of TN and TP in our study area are primarily from urban surface accumulations, which are subsequently transported through surface runoff during extreme rainfall events. In this paper, the pollution risk zoning maps show that terrain factors greatly influence the diffusion of pollutants. Areas with high terrain are more likely to lose pollutants, while areas with low terrain are more likely to accumulate pollutants. However, the results of diffusion are not completely consistent with the terrain distribution, variations in land use, and rainfall intensity, and other aspects all have an impact on the spatiotemporal distribution of NPS pollution [36,49], which requires further exploration.

**Figure 12:**
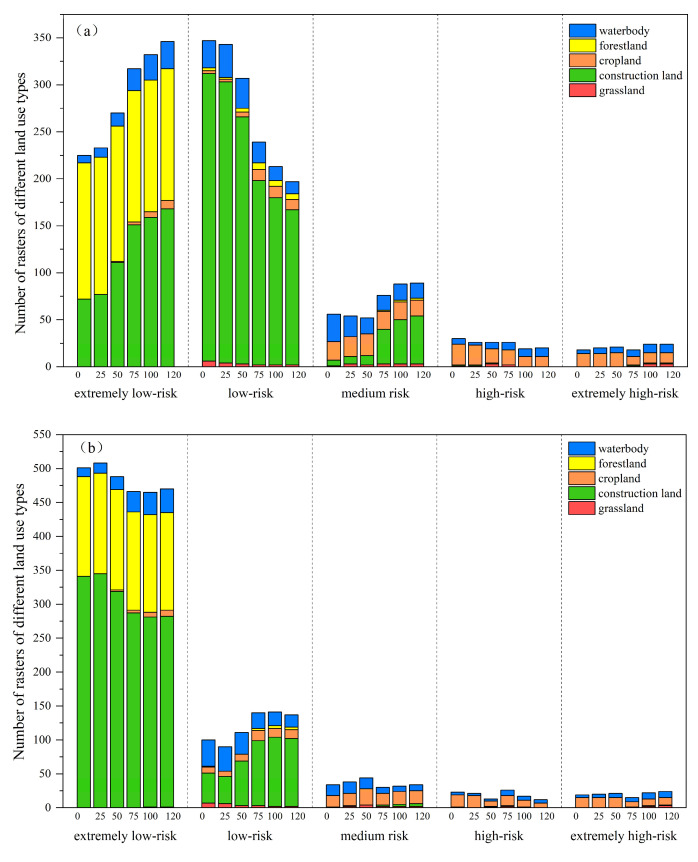
Variations in the quantity of rasters within each risk area of TN (**a**) and TP (**b**).

During the 120 min diffusion phase, Figure 12 shows variations in the quantity of rasters within each risk area. From the overall distribution point of view, the extremely low-risk areas and low-risk areas of TN and TP are mainly distributed in grids with attributes of forest land, construction land, and water bodies, while the medium-risk areas are mostly spread in grids with attributes of construction land, cropland, and water bodies, and the high-risk areas are primarily spread with rasters, attributes of which are cropland and water bodies. From this, it can be seen that the overall pollution risk of forest land and grassland is low, while the overall pollution risk of cropland and water bodies is high.

1. Forest land: Analyzing the change characteristics of pollution risk, the forest land raster is mainly distributed in extremely low-risk areas, while a small amount of which is distributed in lower-risk areas, and a very small number is distributed in medium-risk areas. It can be seen that the pollutant risk of the forest land is very low, and combined with the fitting curve analysis of the forest land, the pollutants in the forest land are still being lost under extreme rainfall, so the forest land grid continues to maintain a low-risk state during the diffusion process.
2. Construction land: The pollution risk of the construction land grid is mainly distributed in the extremely low-risk area and low-risk area. Among them, the TN load in the construction land grid shifts from the low-risk area to the extremely low-risk area and the medium-risk area, while the TP load is slightly different, showing that the number of grids in the extremely low-risk area decreases and shifts to the low-risk area. Combined with the fitting curve of construction land load, the change in pollution risk in construction land is not significant. Since the number of construction land grids is the largest and the built environment elements in construction land are complex, the uncertainty in the diffusion process is also greater, which may also be the reason why the load change trends in TN and TP are different.
3. Cropland: It can be seen that the number of cropland grids in extremely low-risk areas and low-risk areas gradually increases over time, and the pollution risk gradually shifts from high-risk areas to low-risk areas. As shown in the fitted curve of cultivated land, the risk in cultivated land is continuously decreasing.
4. Grassland: Although the fitting curve of grassland shows that the pollution load in grassland increases monotonically and has the largest change rate, this result needs further verification in subsequent research for the small number of grassland grids from the local analysis.
5. Water body: Observing Figure 12, we can see that the number of water grids in both the extremely low-risk area and the extremely high-risk area is increasing, which means that the risk in some grids increases, while the risk in some grids decreases. However, this is different from the monotonically rising trend of the water body fitting curve. Judging from the water body fitting curve, the pollution load in the water body grid will continue to rise, which is consistent with the increasing number of water body grids in medium- and high-risk areas. However, due to the large raster area, some rasters contain a variety of land use types, which may impact the changes in risk. Some water body rasters located in low- and medium-risk areas contain a small amount of forest land, cultivated land, construction land, and other land types, while the risk of pollution in forest land and cropland tends to decrease, which may lead to a reduction in the risk in the grid.

These research results demonstrate how different land uses affect the dynamic diffusion process, which is crucial for rationally regulating the diffusion risk of urban surface pollutants in the changing environment in the future.

Additionally, from the dynamic diffusion process of NPS pollution, we can know that with the occurrence of extreme rainfall, surface pollutants migrate and diffuse to a greater extent along with surface runoff, and the changes in the diffusion rate first increase and then decrease. Considering that the total amount and intensity of rainfall events greatly influence the spread of NPS pollution [25], there exists a positive correlation between rainfall severity and the NPS pollution loss. The causes of the development of the spatiotemporal distribution features of NPS pollution were also examined. Figure 3 illustrates that the rainfall intensity first increases and then decreases, and it reaches its greatest value at 49 min. The NPS pollution diffusion state varies most before and after 50 min, coinciding with the rainfall intensity change process. That is to say, there is a positive correlation between the change rate of NPS pollution spatial distribution and the change rate of rainfall intensity.

It is important to note that our simulation did not incorporate the specific physicochemical properties of different contaminants, which could potentially affect their diffusion rates and risks under extreme rainfall conditions. In future work, we plan to extend our model to incorporate more detailed chemical processes, such as pollutant degradation, adsorption/desorption, and precipitation/dissolution, which could provide a more comprehensive understanding of pollutant behavior in the water environment.

## 5. Conclusions

This study aims at the serious problem of NPS pollution during heavy rains. By fusing the CA model and GIS technology, combined with an SW2D-GPU model, the study systematically explores the migration and diffusion mechanism of urban NPS pollution under extreme rainfall and its impact on land use changes, accurately detecting the changes in pollution risk at each diffusion stage. The research results are as follows:

(i) Under extreme rainfall scenarios, the distribution status of NPS pollution becomes more and more dispersed. The distribution area of high-risk and extremely high-risk grids has increased, and the average pollution load and maximum pollution load of high-risk areas increased greatly. The diffusion rate of NPS pollution is positively correlated with rainfall intensity. The heavier the rainfall, the stronger the erosion of pollutants, and the faster the pollution diffusion rate.
(ii) Pollutant diffusion is significantly influenced by land use type: the pollution risk in forest land and cropland is declining and the pollutant loss rate in forest land is greater. The pollution load in waters continues to accumulate, and the pollution risk continues to increase.
(iii) The pollution risk zoning results obtained through the simulation method in this paper can provide targeted governance suggestions for urban storm and flood management. This method can capture key areas of violent growth in pollution concentrations, thereby effectively providing information to environmental management practitioners, and helping to strengthen policy formulation in pollution control. At the same time, the analysis of the responses of different land use types to non-point source pollution under heavy rains can provide valuable insights for urban planning managers in planning land use and avoiding environmental pollution.

Compared to previous research, this study realizes the refined simulation of NPS pollution diffusion in areas where detailed data (such as actual monitoring data, river official website data, road pipe network data, and so on) are missing, bridging the gap in scale selection and model application, which were mostly employed in previous studies. However, this study does not consider state changes of pollutants in water, nor does it characterize the diffusion process of pollutants in a continuous manner. This will affect the accuracy of the NPS pollution diffusion simulation results and requires further improvement in future research.

## Figures and Tables

**Table 1:** The pollution export coefficients.

	Grassland	Cropland	Construction Land	Forest Land	Water Body
TN	1.00	2.90	1.10	0.24	1.50
TP	0.02	0.09	0.02	0.02	0.04

**Table 2:** Results of sensitivity analysis.

Parameter	Value Setting	Maximum Depth (m)	Inundation Area (km^2^)	Baseline Scenario Parameter
Manning’s Coefficient	0.01	4.452	116.459	0.012
	0.02	4.438	117.589	
	0.03	4.419	117.593	
Permeability	0.3	4.569	11.589	0.5234
	0.5	4.453	116.954	
	0.7	4.428	114.892	
Iteration Time Step (s)	0.02	4.412	114.694	0.04
	0.06	4.536	115.558	
	0.08	4.539	115.694	
Grid Refinement (m)	5	4.442	116.062	5
	20	3.864	102.489	
	50	3.156	98.544	

**Table 3:** Initial pollution risk zoning for TN.

Risk Zoning	Pollution Load Range (kg/a)	Area Proportion
Extremely low-risk	(8476.44, 207,310.26)	33.48%
Low-risk	(207,310.26, 567,696.57)	51.78%
Medium-risk	(567,696.57, 1,251,187.84)	7.85%
High-risk	(1,251,187.84, 2,133,512.93)	4.29%
Extremely high-risk	(2,133,512.93, 3,177,390.51)	2.67%

**Table 4:** Initial pollution risk zoning for TP.

Risk Zoning	Pollution Load Range (kg/a)	Area Proportion
Extremely low-risk	(706.37, 7617.12)	74.11%
Low-risk	(7617.12, 20,286.83)	14.94%
Medium-risk	(20,286.83, 41,786.94)	4.88%
High-risk	(41,786.94, 64,054.82)	3.25%
Extremely high-risk	(64,054.82, 98,608.67)	2.81%

## Data Availability

The data will be made available on request.

## Abbreviations

The following abbreviations are used in this manuscript:

NPS	Non-point source
SW2D-GPU	A two-dimensional shallow water model accelerated by a general-purpose graphics processing unit
L-THIA	Long-Term Hydrologic Impact Assessment
SWAT	Soil and Water Assessment Tool
WEPP	Water Erosion Prediction Project
SWMM	Storm Water Management Model
TN	Total nitrogen
TP	Total phosphorus
CA	Cellular automata
